# The Gut Bacterial Community of *Chlaenius pallipes* (Coleoptera: Carabidae) Associates with Their Habitat and Morphology

**DOI:** 10.3390/insects13121099

**Published:** 2022-11-29

**Authors:** Yuno Do, Jun-Kyu Park, Woong-Bae Park, Min-Seob Kim

**Affiliations:** 1 Department of Biological Science, Kongju National University, Gongju 32588, Republic of Korea; 2 Environmental Measurement & Analysis Center, National Institute of Environmental Research, Incheon 22689, Republic of Korea

**Keywords:** carabid beetle, indicator, landmark morphometrics, stable isotope

## Abstract

**Simple Summary:**

This study aimed to determine i the gut microbiome could be used as an indicator of an organism’s health or the health of the population using the *Chlaenius pallipes* (Carabid beetle) as a model organism from the terraced paddy fields and the large-sized paddy fields. The composition of the gut bacterial community associated with habitat types. Gut bacterial community of *C. pallipes* differed in diversity, similarity, and composition between the habitat conditions. Additionally, food resource quality derived from the stable nitrogen isotope ratio was significantly correlated with richness of the gut bacterial community.

**Abstract:**

We investigated whether the gut bacterial community of *Chlaenius pallipes* could represent the health conditions of individuals or populations based on where these beetles inhabit. Considering the ecological traits of the species, the gut bacterial communities of carabid populations inhabiting stable or unstable habitats were compared. Food resource quality (δ^15^N) and morphological shape, especially body and wing size, may be significant factors that directly or indirectly affect the gut bacterial community of carabid beetles. Firmicutes (51.7%) and Proteobacteria (36.3%) were the predominant phyla in the gut bacterial community of *C. pallipes*. A significant difference in the gut bacterial community structure was observed between organisms inhabiting unstable and stable habitats in this study. Wing size, as estimated by centroid size, was correlated with differences in the gut bacterial community composition of the species. Even if a factor is not strong enough to determine the survival of carabid beetles, the composition of the gut bacterial community can change. We found that although each individual has a large variation in the gut bacterial community composition, the gut bacterial community can be used to evaluate the condition of each habitat through consistent investigation. Habitat assessment based on changes in the number of carabid beetle species and their composition requires relatively long-term research; however, the gut bacterial community of carabid beetles can help identify short-term environmental changes.

## 1. Introduction

Carabid beetles are frequently used as indicators of biodiversity and habitat quality. Diverse and abundant carabid beetles are distributed over a broad geographic range and are well known, both taxonomically and ecologically [1]. Carabid beetles are sensitive to environmental changes in their habitat environments; changes involving land use, management practices, urbanization, and climate change, on local to global scales can have great effects on this population. The carabid beetle’s response to environmental changes is frequently identified by an increase or decrease in the number of species and the proportion of species that prefer specific habitats (e.g., forest, grassland, or dryland) [2]. Therefore, studies using carabid beetles as an indicator species typically compare carabid beetle communities and assemblages in different environmental habitats, such as in disturbed and undisturbed habitats, based on the ecological traits (habitat preference, food preference, and flight capacity) of the species [2,3]. However, it is difficult to assess habitat conditions, resulting in difficulties identifying the community structure causing the low diversity of carabid beetle species. Moreover, ecological knowledge is required to determine the health conditions of rare species [4]. Thus, identification of a measurement that can help identify the response to environmental changes at the individual and population levels of carabid beetles may resolve these challenges [5,6,7].

Many studies on the gut microbiome have been conducted not only in humans, but in various other species [8,9]. Analyses of the gut microbiome can aid in identifying the differences between the gut microbiome in individuals under normal conditions and those under abnormal conditions to assess the impact of the gut microbiome on metabolic disorders [10]. The community structure and metabolic activity of the gut microbiome are affected by the feeding behavior, habitat preference, and habitat conditions of insects [11,12]. The insect’s gut bacteria serve important roles in the digestion, detoxification, development, pathogen resistance, physiology, and behavior of the host [13]. Although the relationship between gut bacteria and insect health has yet to be fully elucidated, gut bacteria play a vital role in food uptake and in providing nutrients that are essential for the survival and reproduction of insects [14]. Gut bacterial diversity is significantly reduced by factors that threaten insect health, such as pesticides and extreme environmental conditions [15]. Taken together, the diversity and structure of the gut bacterial community may serve as a proxy for an insect’s health.

In this study, we investigated whether the gut bacterial community of carabid beetles could represent the health conditions of each individual or at the population level. Considering the ecological traits of each species, the *Chlaenius pallipes* were used to assess the health of the gut bacterial community at the population level. *C. pallipes* is a hygrophilous species that is found in a wide range of habitats, including forests, grasslands, farm fields, paddy fields, and riparian areas. However, even within the same habitat type, the population density of carabid beetle species varies depending on the surrounding landscape complexity and habitat management practices. We investigated *C. pallipes* in two different types of paddy fields: a large paddy field (LP) and a terraced paddy field (TP). The LP is a consolidated paddy field used for the mechanization of farming and it is conventionally managed. The TP that was not enlarged by land consolidation is located on sloping hillsides and is surrounded by a forest. *C. pallipes* inhabits both types of paddy fields, but the TP supports spatial heterogeneity and a more stable habitat for *C. pallipes* than the LP, which is a spatially homogeneous and unstable habitat.

Furthermore, we measured food resource quality and morphological shape, especially body and wing size, which may be significant factors that directly or indirectly affect the gut bacterial community of carabid beetles. The gut bacterial community of a species is distinguished by phylogeny but is most affected by food sources. The stable nitrogen isotope ratio (δ^15^N) can be used as a measurement to determine the quality of food resources. Although it is known that *C. pallipes* predates insect larvae, snails, and earthworms when preying on various protein sources, it has a wide range of δ^15^N sources so δ^15^N is also increased [16,17,18]. The body and wing sizes of *C. pallipes* may also be sensitive to habitat conditions [19,20,21]. In general, highly disturbed areas support carabid assemblages with species of smaller sizes than those with less disturbed sites. In addition, body size decreases in disturbed habitats [22]. Although a few studies have suggested that the wing development of carabid beetles is not influenced by temperature and food supply at the individual level [23], higher activity in individuals with large wings can lead to a more diverse diet because they are more likely to encounter other types of prey [24].

In this study, we tested the assumption that when *C. pallipes* is present in the TP environment, the stable habitat will cause the species to have more diverse gut bacteria. We also expect that this environment will be associated with a change in *C. pallipes*’ morphological shape and affect the food resources of the species.

## 2. Materials and Methods

### 2.1. Carabid Beetle Sampling, Measurement, and Dissection

**Figure 1:**
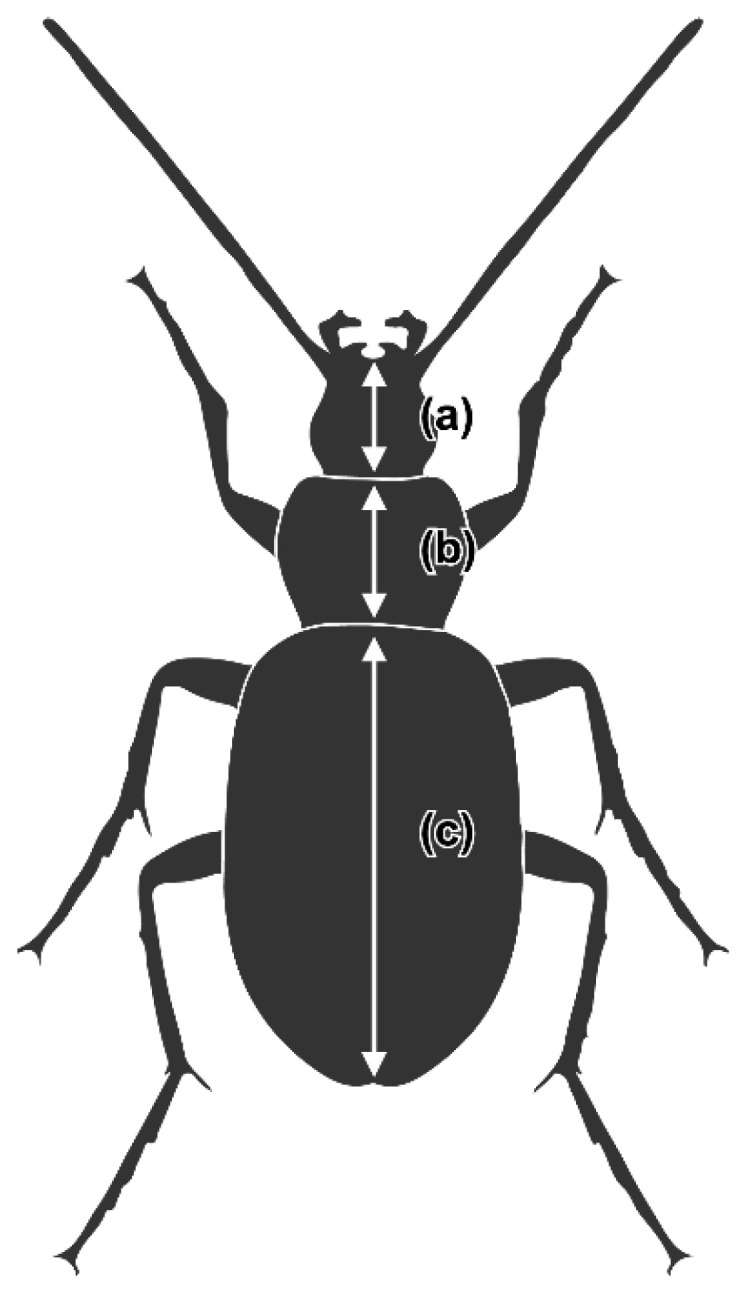
Each parameter for measuring body length of *C. pallipes*. (**a**) elytra length (the posterior end of the scutellum to the terminus of the right elytron); (**b**) pronotum length (the central furrow pronotum); (**c**) head length (the labrum and juncture of the occiput to postgena).

Thirty-two male *C. pallipes* species were collected after 8 p.m. between May and June 2021 from the LP and TP in Gongju-si, Chungcheongnam-do, South Korea. We collected live specimens inhabiting herbaceous banks by hand in both the LP and TP. The specimens were then transported to the laboratory facility at Kongju National University and were stored individually in 15 mL conical tubes at −80 °C. The day after collection, we photographed the specimens using a digital stereo microscope (Ash Technologies Omni-Core Digital Microscope). Body length was calculated by combining the elytra length as the distance between the posterior end of the scutellum and the terminus of the right elytron, pronotum length was measured along the central furrow pronotum, and head length as the distance between the labrum and juncture of the occiput and postgena (Figure 1); lengths were measured from specimen photographs using the free software, ImageJ version 1.8.0 [25]. The right hind wing of each specimen was carefully removed for geometric morphometric analysis. Each specimen’s midgut was dissected under sterile conditions on a clean laminar-flow bench. Dissected midguts were placed individually in a sterile 2 mL micro centrifuge tube and then stored at −80 °C until DNA extraction. Remaining specimens, including the head, pronotum, elytron, and legs, were also placed individually in a sterile 2 mL micro centrifuge tube and then stored at −80 °C until stable isotope analysis was performed.

### 2.2. DNA Extraction, PCR, and Next Generation Sequencing

Genomic DNA from the intestinal microorganisms of the beetles were extracted from midgut samples using a Qiagen DNeasy Powersoil Pro Kit (Qiagen, Hilden, Germany). Briefly, after adding lysis buffer to the sample, the sample was homogenized for 5 min at a speed of 30 m/s using a TissueLyser II (Qiagen, Hilden, Germany), and then genomic DNA was obtained according to the manufacturer’s protocol. The extracted DNA was quantified on a DeNovix-QFX fluorometer (Denovix Inc., Wilmington, DE, USA) using a QFX dsDNA High Sensitivity Assay Kit (Denovix Inc., Wilmington, DE, USA). The quality of genome DNA was confirmed through electrophoresis, and it was confirmed that all samples were not abnormal.

The extracted genomic DNA of the microorganisms from beetle midguts were used to amplify the 16s ribosomal RNA (16s rRNA) region. We used barcoded PCR primers with the Illumina overhang adapter sequence 515F (5′—TCGTCGGCAGCGTCAGATGTGTATAAGAGACA GGTGCCAGCMGCCGCGGTAA—3′) and 806R (5′—GTCTCGTGGGCTC GGAGATGTGTATAAGAGACAG GGACTACHVGGGTWTCTAAT—3′) to amplify the V4 region of the 16s rRNA gene. The amplified PCR product was purified using the Agencourt AMPure XP PCR purification system (Beckman Coulter, Brea, CA, USA), and index PCR was performed using the Nextera XT Index Kit (Illumina, San Diego, CA, USA). The indexed PCR product was purified using the Agencourt AMPure XP PCR purification system. Finally, the concentration of the second amplified PCR product was diluted to 10 nmol/L using Qubit and then diluted again to 1 nmol/L before pooling the samples. Pooled samples were analyzed using an Illumina Miniseq system (Illumina, Inc., San Diego, CA, USA).

Raw paired-end FASTQ reads were processed using QIIME2 (v.2022.2) and its associated plugins. The DADA2 plugin was used for quality filtering, denoising, paired-end merging, and feature-table construction. The trimming parameters were determined based on the demux visualization. The taxonomic assignment of appropriate taxa was performed using the default settings of the feature-classifier plugin for QIIME2 against the EzBioCloud 16S reference database (https://www.ezbiocloud.net/, accessed on 1 August 2022). The dataset was filtered to contain only bacteria.

### 2.3. Landmark-Based Geometric Morphometrics

**Figure 2:**
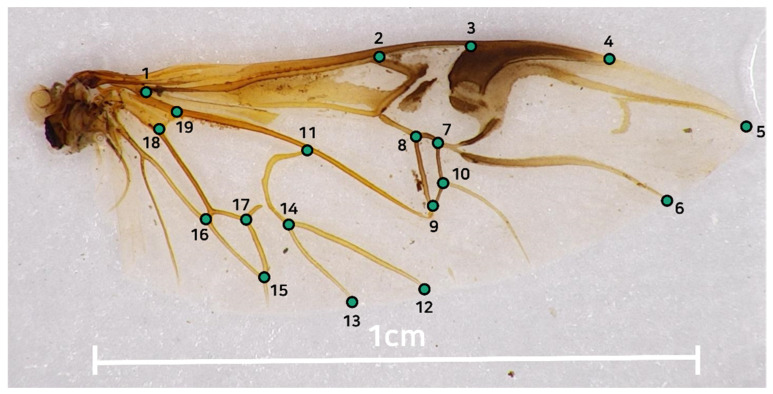
Locations of 19 landmark points to visualize the wing shape in the right hindwings of *C. pallipes*.

Photographs of the detached hind wings were also taken with a digital stereo microscope (Ash Technologies Omni-Core Digital Microscope). Eighteen landmarks were manually measured by one person from each image using the TpsDig2 software (Figure 2). Landmark coordinates were superimposed using full Procrustes fit, which involves translation, rotation, and scaling of landmarks using MorphoJ [26]. Canonical variate analysis (CVA) as a multivariate analysis of wing shape was conducted on all landmark coordinates to compare morphological differences in wing shape between paddy field types. The differences in wing shape obtained from the different paddy field types were visualized using canonical variate axes. Statistical analysis of the differences in wing shape between paddy field types were performed using the Mahalanobis distance of CVA and the *p*-value of the distance. Wing size was estimated based on its centroid size.

### 2.4. Nitrogen Stable Isotope Ratio Analyses

The remaining specimens, including the head, pronotum, elytron, and legs, were freeze dried and ground. The untreated samples were used for nitrogen isotope analysis. Stable nitrogen isotope ratios of the samples were measured using an elemental analyzer coupled with an isotope ratio mass spectrometer (EA-IRMS; EuroEA-Isoprime IRMS, GV Instruments, UK). Stable isotope ratios were calculated using standard δ notation:

δX (‰) = ((Rsample/Rreference) − 1) × 1000

Here, X = N (nitrogen) and R is the corresponding ratio of ^15^N/^14^N. The standard reference material was Vienna Pee Dee Belemnite (VPDB) for atmospheric nitrogen (N_2_). The analytical precision was 0.05‰ to 0.1‰ for nitrogen.

### 2.5. Statistical Analysis

The alpha and beta diversity analysis of bacterial communities were conducted using the microeco package in R4.2.0 [27]. Significant differences in diversity indices between the TP and LP were measured using a t-test analysis. Bacterial taxonomic (ASVs) data were Hellinger transformed, and Bray–Curtis distance measures were used to generate dissimilarity matrices. Differences in bacterial community composition were visualized using principal coordinate analysis (PCoA). The differences in Bray–Curtis dissimilarity in bacterial communities between TP and LP environments were assessed using a *t*-test.

The significant the nitrogen isotope ratio was measured and statistical analyses were performed using the vegan package in R4.2.0. This package was also used to analyze the centroid size of the carabid beetle’s hind wings and to analyze functional differences between groups.

We used Kendall’s rank correlation coefficient (T) to test for correlations between the diversity indices for bacterial communities of each sample (such as observed species, Chao1, Shannon diversity index, and Pielou’s evenness) and morphological data (such as body length and the centroid size of the hind wing of the carabid beetle). The Kruskal–Wallis rank sum test was used to assess differences in the bacterial community composition at the class level. Redundancy analysis (RDA) was employed to determine the relationship between bacterial community composition and environmental factors (δ^15^N(‰), body length, and centroid size of the hind wing) using the microeco package in R.

## 3. Results

### 3.1. Difference in C. pallipes Food and Morphological Shape according to Habitat Types

**Figure 3:**
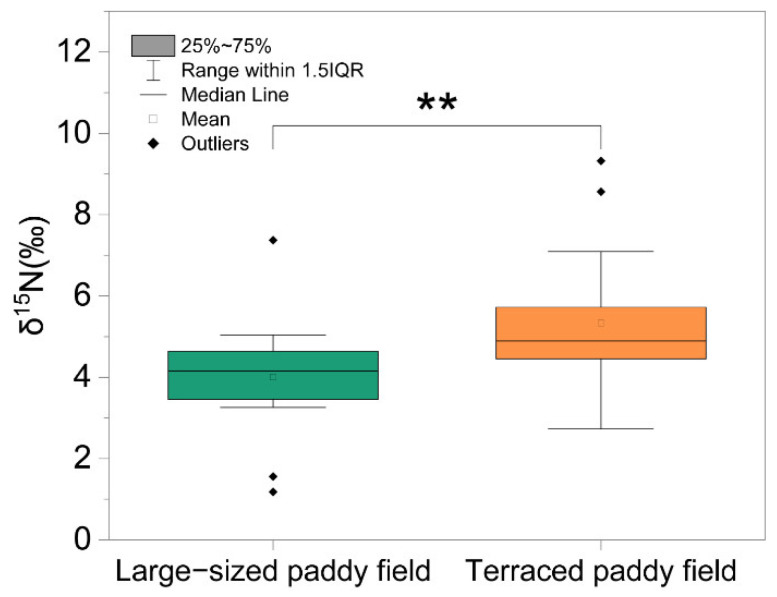
The nitrogen stable isotope ratio (δ15N) for *C. pallipes* in large-sized paddy field and terraced paddy field. Asterisk indicates a significant difference at ** *p* < 0.01 based on t-test.

To begin to determine if a biomarker for *C. pallipes* overall health can be elucidated, we first measured the stable nitrogen isotope ratio, which is used as a marker of dietary protein sources. The stable nitrogen isotope ratio of *C. pallipes* in the TP environment ranged from 4.45 to 5.72, while the ratio of *C. pallipes* in the LP environment varied from 1.18 to 7.37. *C. pallipes* had a significantly higher stable nitrogen isotope ratio in the TP environment, compared to that of the LP (*t*-test *p* > 0.01) (Figure 3).

**Figure 4:**
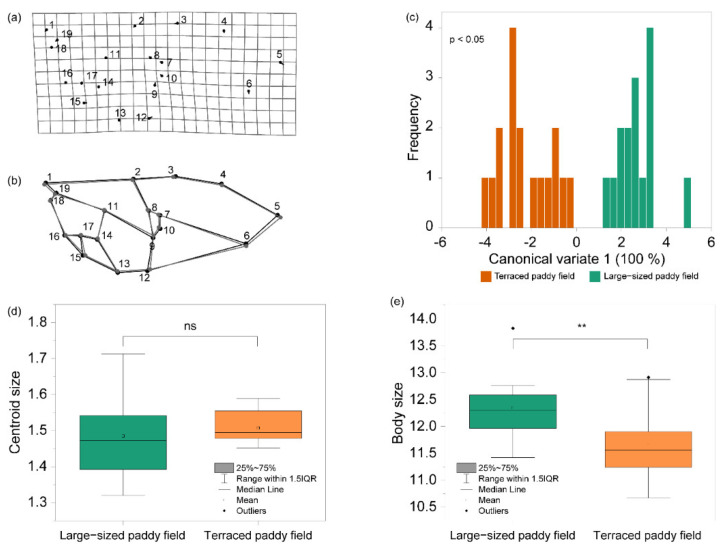
The variation of wing shape and body size of *C. pallipes* between the large-sized paddy field (LP) and terraced paddy field (TP). (**a**) Deformation grid and lollipop graph showing morphological variation in canonical variate 1 (CV1). (**b**) Wireframe graph illustrating minor morphological difference based on CV1. (**c**) Histogram consisting of canonical variate 1 shows that *C. pallipes* in LP and TP have different wing shape. Wing shape significantly grouped according to CV1 by calculating Mahalanobis distance in CVA. (**d**) Comparison of centroid size as a proxy for wing size of *C. pallipes* between LP and TP. (**e**) Comparison of body size of *C. pallipes* between in LP and TP. The *p* value in (**d**,**e**) indicates the result of the comparison with *t*-test, where ** *p* < 0.01, and ns “not significant”.

Next, the morphological shape was measured. Circles in the deformation grids and grey wireframe graphs indicate the wing shape of individuals with the lowest CV values (Figure 4a,b). The wing shape CVA explained 100% of the variance with the first canonical axis (Figure 4c). The positive end of CV1 showed the individuals that were grouped in the LP environment, whereas those individuals in the TP environment were grouped toward the negative CV1 axis (Figure 4c). The wing size, which was estimated by its centroid size, was not significantly different (*t*-test, *p* > 0.05) between the beetles from the LP and TP environments (Figure 4d). However, body size was significantly larger in the TP group than in the LP group (Figure 4e) (*t*-test, *p* < 0.01)

### 3.2. Effect of Habitat Type on Gut Bacterial Community Composition

**Figure 5:**
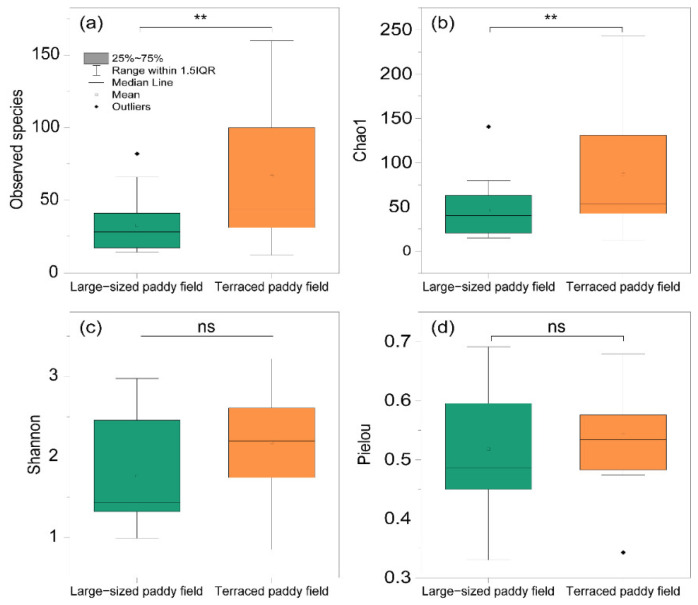
Alpha diversity of gut bacterial community of *C. pallipes* according to the (**a**) Observed species, (**b**) Chao1, (**c**) Shannon index, and (**d**) Pielou’s evenness at OUT level represented as boxplot. The *p* value shown above each graph indicates the result of the comparison with t-test, where ** *p* < 0.01, and ns “not significant”.

**Figure 6:**
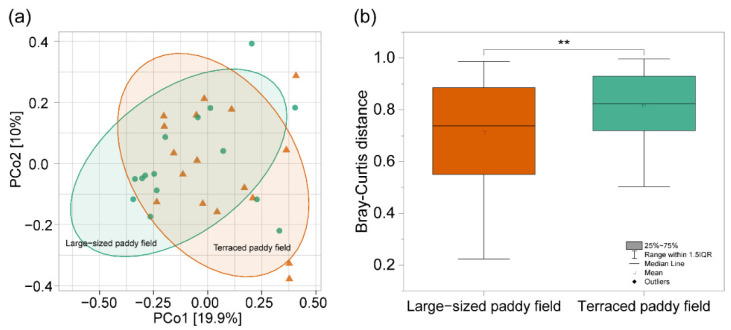
The difference of the gut bacterial community structure of *C. pallipes* between the terraced paddy field and the large-sized paddy field. (**a**) Principal coordinate analysis (PCoA) plot based on Bray–Curtis dissimilarity. (**b**) The dissimilarities of gut bacterial communities between the terraced paddy field and the large-sized paddy field. Asterisks indicate a significant difference at ** *p* < 0.01 in (**b**) based on *t*-test.

Analysis of the *C. pallipes* gut microbiome resulted in species richness (observed species and Chao1) in the gut bacterial communities of *C. pallipes* from TP environments; the number of bacterial species was significantly higher in the TP group than those in the LP (*t*-test *p* < 0.05, Figure 5a,b). However, there was no significant difference between the evenness (Shannon diversity index and Pielou’s evenness) of the TP and LP groups (*t*-test *p* > 0.05, Figure 5c,d). Although PCo1 and Pco2 contributed to only 29.9% of the variance (Figure 6a), significant differences in the bacterial community composition between the TP and LP groups were found via PCoA analysis (Bray–Curtis dissimilarity *t*-test *p* < 0.05, Figure 6b).

**Figure 7:**
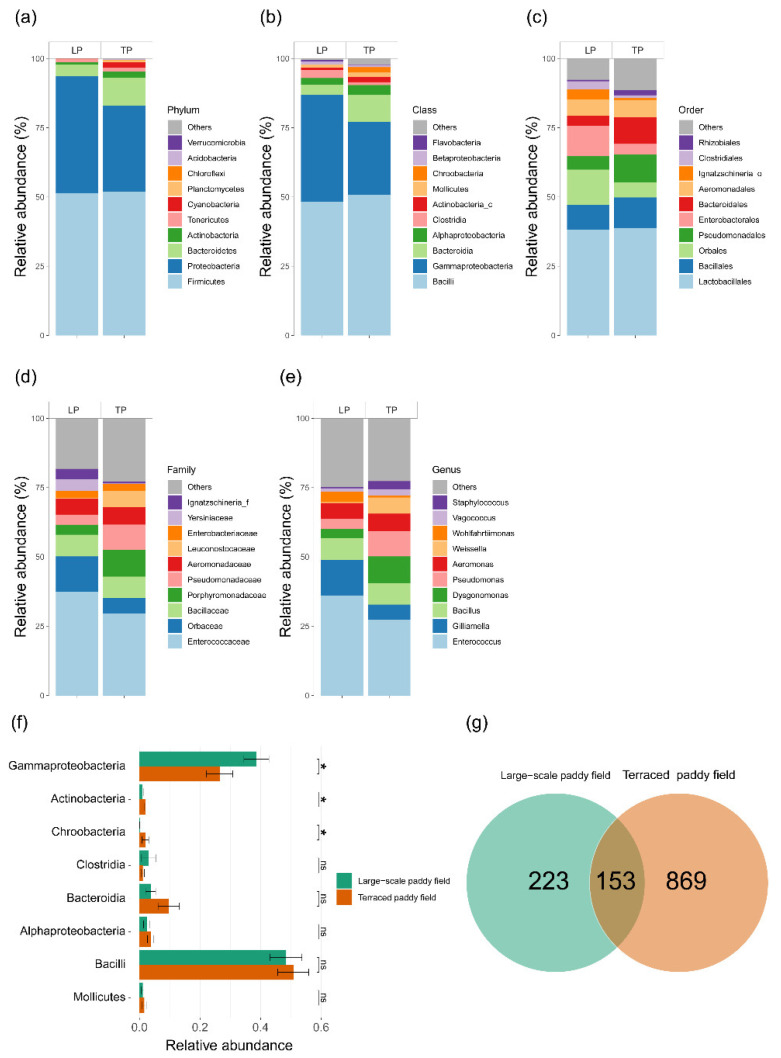
Relative abundance of gut bacterial taxa of *C. pallipes*. Bacterial community structures at phylum (**a**), class (**b**), order (**c**), family (**d**), and genus (**e**). (**f**) Kruskal–Wallis rank sum test performed at class rank for the gut bacterial taxa. (**g**) Venn diagram illustrating the overlap of gut bacterial OTUs. LP and TP in (**a**–**e**) indicates large-scale paddy field and terraced paddy field, respectively. The *p* value in (**f**) indicates the result of the comparison with Kruskal–Wallis rank sum test, where * *p* < 0.05, and ns “not significant”.

The gut bacterial community is mainly dominated by Firmicutes, Proteobacteria, and Bacteroidetes. In the gut bacterial community of *C. pallipes* from the TP environment, the proportion of Bacteroidetes was higher than that from the LP, while the proportion of Proteobacteria was higher in the LP group (Figure 7a). At the class level, the proportion of Gammaproteobacteria in the LP group (36.4%) was significantly higher than in the TP group (26.9%) (Kruskal–Wallis test, *p* < 0.05, Figure 7b). Accounting for the lower proportion of bacterial communities, Acidobacteria (TP 1.8%, LP 0.6%) and Chroobacteria (TP 2.4%, LP 0.2%) were significantly higher in the TP group than in the LP group. A total of 153 gut bacterial Operational Taxonomic Units (OTUs) (12.2% of the total OTUs) of *C. pallipes* overlapped between the LP and TP groups (Figure 7c). There were 223 (17.9%) and 869 (69.8%) gut bacterial OTUs of *C. pallipes* that were specific to LP and TP, respectively.

### 3.3. Correlation among Diet, Morphological Shape, and Bacterial Community

**Figure 8:**
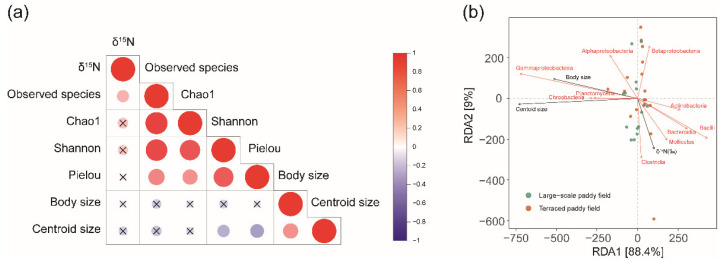
Correlation among bacterial communities among environmental factors. (**a**) Correlation among diet (nitrogen stable isotope ratio, δ^15^N), gut bacterial species richness (Observed species and Chao1), species evenness indices (Shannon index and Pielou’s evenness), and morphological characteristics (body size and wing size of *C. pallipes*) by Kendall’s correlation test. The circles with “X” in the correlation plot indicate “not significant”. The color gradient of correlation plot indicates the Kendall tau correlation coefficient. (**b**) Redundancy analysis (RDA) indicating the relationship between bacterial community structure and environmental factors.

Significant positive correlations were obtained by Kendall’s correlation test for both the stable nitrogen isotope ratio and the observed species richness (τ = 0.25, *p* < 0.01). Centroid size, which is a proxy for wing size, was negatively correlated with the evenness indices, such as the Shannon index (τ = −0.25, *p* > 0.05) and Pielou’s evenness (τ = −0.32, *p* > 0.05) (Figure 8a).

RDA at the class level was performed to understand the main environmental factors, such as δ^15^N(‰), body length, and centroid size of the hind wing, which contributed to changes in the bacterial community structure (Figure 8b). Centroid size (r^2^ = 0.27, *p* < 0.01) was the strongest among the experimental factors that represented the coefficient of determination for bacterial species distribution. Centroid size was positively correlated with Chroobacteria and Planctomycetia, and body size was associated with Gammaproteobacteria. Interestingly, δ^15^N was positively correlated with Clostridia and negatively correlated with Alphaproteobacteria presence.

## 4. Discussion

This study indicated that the gut bacterial community of carabid beetles may be significantly affected by habitat conditions at the population level. Thus, even if an environmental factor is not strong enough to affect the survival of carabid beetles, the composition of the gut bacterial community can change.

In general, macropterous individuals occupy disturbed habitats, and the wing length of brachypterous individuals is longer when inhabiting disturbed habitats [28]. Active changes in wing size are observed in species with wing dimorphisms, which are determined by environmental changes, such as temperature, high food supply, and habitat disturbance [29]. However, in this study, we did not find a significant difference in wing size between unstable and stable habitats, as wing dimorphism was not found in *C. pallipes* [30]. Nevertheless, we only found a significant difference in the morphological wing shape between beetles inhibiting stable and unstable environments. This suggested that habitat conditions may cause delicate changes in wing morphological shape, not only wing size.

The body size of *C. pallipes* from the unstable habitat was significantly larger than from the stable habitat. Body size may be positively correlated with sufficient food supply and food quality [31,32]. However, the relationship between dietary intake and body size may depend on the species. This data agrees with previous work showing that the body size of carabid beetles in disturbed habitats is larger than that in undisturbed habitats [33].

Individuals with sufficient nutrient intake may have increased δ^15^N and body size; however, in this study, contradictory results were found between body size and δ^15^N. Even if habitats are different, in stable habitats that support sufficient food sources, habitat generalists (rather than specialists who prefer only certain prey and habitats) may have no difference in gut bacterial community structure or diversity depending on their habitat [34].

The significant difference in the gut bacterial community between unstable and stable habitats in this study may be the result of long-term effects of complex correlations among carabid beetles, food resources, and the environment. In a previous study, the bacterial community structure of carabid beetles was not changed by short-term perturbations in food resources [35]. These results suggest that carabid beetles may have a persistent gut bacterial community, and microbiome variability may be affected by long-term changes to host food resources or the environment. The δ^15^N measured from the head, pronotum, elytron, and legs of the carabid beetles in this study supported this result. Although the difference in δ^15^N between the LP and TP groups did not show a significant difference in trophic level (increase of 2.5‰), the difference in δ^15^N may be associated with gut bacterial diversity due to long-term environmental impacts. This is because the exoskeleton and muscle of insects reflect the isotopic composition of resources incorporated during development [17]. In a previous study, gut bacterial community structure and diversity were clearly distinguished according to feeding habits (i.e., carnivores, herbivores, omnivores, and scavengers) rather than according to habitat [36]. Feeding habits that emerge as a result of specialization and adaptation to the environment are closely related to phylogenesis and are found to affect gut bacterial communities more strongly than differences in habitats. However, at the population level, differences in the habitat environment can also induce changes in gut bacterial communities.

Proteobacteria and Firmicutes were the predominant phyla in the gut bacterial community of *C. pallipes*. This result is consistent with that of a previous study showing that these phyla are generally associated with the insect gut [12]. The abundance of diverse Firmicutes dominates carnivorous insects and shows differences between microbial communities of herbivorous or omnivorous species [37]. At the class level, a higher diversity and abundance of Gammaproteobacteria was recorded in unstable habitats (LP) compared to stable habitats (TP) in this study. An imbalance in diet could drive an increase in the proportion of Gammaproteobacteria in carabid beetles. The Gammaproteobacteria ratio increased significantly in individuals fed artificial food in the laboratory [38]. In herbivorous insects, starvation in locusts is associated with an increase in Gammaproteobacteria [39]. Honeybees exposed to parasites and insecticides also exhibit a high Gammaproteobacteria ratio [40].

The number of species and community structure of the gut bacterial community of *C. pallipes* showed a difference between unstable and stable habitats, but the diversity indices based on evenness were not significantly different in either habitat. This indicated that the environmental differences between the two habitats did not induce a particular bacterial taxon to dominate. Although the composition ratio of Gammaproteobacteria is high in unstable habitats, it does not seem to have been dominant enough to alter the diversity of the gut bacterial community individually. This is a natural result because the environmental difference between these habitats is not an extreme one, such as exposure to the pesticides or parasites mentioned above, or artificial food source treatment.

## 5. Conclusions

The results of the present study suggest that the gut bacterial community of carabid beetles may be a good indicator of subtle environmental differences. Although each individual has a large variation in gut bacterial community composition, the gut bacterial community can be used to evaluate the condition of each habitat through consistent investigation. Habitat assessment based on changes in the number of carabid beetle species and their composition requires relatively long-term research; however, the gut bacterial community of carabid beetles can help identify short-term environmental changes.

## Data Availability

Data generated in this study are available upon reasonable request to the corresponding author.
